# Online Doc: Is Social Media Education a Tool for Melanoma Prevention? A Survey-Based Analysis Among Romanian Digitally Active Users

**DOI:** 10.7759/cureus.50328

**Published:** 2023-12-11

**Authors:** Luana-Andreea Boșoteanu, Mariana Așchie, Mădălina Boșoteanu

**Affiliations:** 1 Institute of Doctoral Studies, Doctoral School of Medicine, Ovidius University of Constanta, Constanta, ROU; 2 Department of Dermatology, Elias Emergency University Hospital, Bucharest, ROU; 3 Department of Pathology, Faculty of Medicine, Ovidius University of Constanta, Constanta, ROU; 4 Clinical Service of Pathology, ”Sf. Apostol Andrei” Emergency County Hospital, Constanta, ROU; 5 Department VIII – Medical Sciences, Academy of Romanian Scientists, Bucharest, ROU

**Keywords:** skin cancer campaigns, sun protection, dermatological awareness, social media, melanoma

## Abstract

Background: In the contemporary era, people are extracting medical information from content-sharing means on the Internet, and the most popular virtual platforms authorize users to access knowledge, guidance, and exchange opinions. Doctors have massively joined online communities and developed educational accounts to meet the informational necessities of general users. The aim of the current study is to identify the role of social media in augmenting the awareness of melanoma.

Methods: For this observational study, questionnaires were disseminated to the general population over eight weeks consecutively, through online channels, represented by Facebook (Meta Platforms, Inc., Menlo Park, California, United States) and Instagram (Meta Platforms, Inc., Menlo Park, California, United States), to extract qualitative data related to the relationship between social media, the epidemiological variables, and melanoma-associated/sun protection behaviors. The inclusion criteria comprised constant Facebook and Instagram users, aged between 18-65.

Results: The study included 221 individuals, primarily aged 26 to 35 (47.1%), mostly females (77.82%). Urban dwellers (>200,000 inhabitants) constituted the majority (71.94%), contrasting with a small rural representation (3.61%). Nearly half actively followed medical educational content online (44.79%), while 12.66% avoided online medical advice. Sun-protective habits were prevalent, with 80.99% using SPF (sun protection factor), 54.29% wearing UV-filter (ultraviolet-filter) sunglasses, and 53.84% avoiding sun exposure between 10 AM and 4 PM. Respondents rated sun-protective measures, with the highest average for avoiding sun exposure during peak hours being 7.3 on a scale of 1 to 10. Dermatological advice from social media influenced behaviors, such as SPF use (50.67%) and sun exposure avoidance (45.7%). Dermatological check-ups were infrequent, with 49.32% sporadically visiting a dermatologist. Dermoscopic evaluations were rare (36.66%), with 27.14% using it preventively. Personal and family history of dysplastic nevi and melanoma were low.

Conclusions: This study provides insights into the dermatological behaviors and education of individuals engaging with social media medical content. The results suggest that constant users of social media harbor a receptive attitude toward dermatological advice provided on specific platforms, hence creating an ideal environment for further dissemination and organization of online skin cancer awareness campaigns.

## Introduction

In the contemporary era, people are extracting medical information from content-sharing means on the Internet, due to the prevalence of quotidian contact with portable, hand-held, and electronic devices. This way, the general population receives information in facile and rapid ways using social media, even though the migration towards these platforms is not currently completely understood from the perspective of audience segmentation [1].

The most popular virtual platforms are classified depending on their primary role: photo-sharing browsers (e.g., Facebook (Meta Platforms, Inc., Menlo Park, California, United States), Instagram (Meta Platforms, Inc., Menlo Park, California, United States)), blogs, microblogs (e.g., Twitter (now X) (X Corp., San Francisco, California, United States)), video-sharing applications (e.g., Youtube (YouTube, LLC, San Bruno, California, United States), TikTok (ByteDance Ltd., Beijing, China), and forums (e.g., Reddit (Reddit Inc., San Francisco, California, United States). They authorize users to access knowledge, get guidance, and exchange opinions [2].

Accelerated digitization precipitated not only the high use of virtual channels from the patient’s point of view but also led to the significant migration of healthcare professionals (HCPs) on social networks. Doctors have massively joined online communities and developed educational accounts to meet the necessities of general users and increase the dissemination of medical information. This fact is supported by the statistical data available at the moment, which shows the presence of 65% of all HCPs in online environments [3].

The aim of the current study is to identify the role of social media in augmenting the awareness of cutaneous and mucous melanoma. Therefore, the most frequent risk factors and malpractices of primary/secondary prophylaxis of malignant melanocytic cutaneous tumors will be extracted. A correlation between different types of social media usage and varying degrees of responsibility regarding the importance of avoiding specific high-risk behaviors will be presented. Thus, we aspire to highlight the methods and principles communicable via online means that are necessary for elevating the number of early melanomas diagnosed among the general population and to discuss the potential improvements that can be brought to the design of online skin cancer awareness campaigns in order to amplify their impact and maximize their reach to the target population, by shifting the paradigm from conventional hospital-based displays of information to the use of more appealing online methods.

## Materials and methods

The study was conducted at the Doctoral School of Medicine, Institute of Doctoral Studies, ”Ovidius” University of Constanta, Constanta, Romania, in accordance with the Declaration of Helsinki and approved by the Ethics Committee of “Ovidius” University of Constanta with approval number 17761/28.11.2022. For this observational study, questionnaires were developed by a team composed of one dermatologist and one pathologist and, finally, reviewed by a third member, represented by a pathologist with a special interest in dermatopathology. It was created by focusing on the potential relationship between social media usage and the personal application of good sun-protective behaviors or notions; moreover, inquiries regarding the personal and/or family history of Clark's nevi (dysplastic nevi) and/or melanoma have been included, because they have been considered as independent factors that increase an individual's self-awareness regarding proper solar prophylaxis. The surveys were disseminated to the general population over eight weeks consecutively, from January to February 2023, through online channels, represented by Facebook and Instagram, in order to extract qualitative data related to the relationship between social media, the epidemiological variables, and melanoma-associated/sun protection behaviors. This exact time of the year was chosen in order to gain more accurate responses and to reach a higher number of participants, due to the fact that studies show an increase in social media engagement during winter months when the weather and the shorter daylight length do not allow for mass participation in outdoor activities [4].

The inclusion criteria comprised constant Facebook and Instagram users, aged between 18-65. If the survey respondent was under 18 or above 65 years old and/or stated that they were not using virtual platforms of any kind, they were excluded from the analysis. These age criteria were chosen because the elderly population is not generally considered representative of the percentage of smartphone ownership; hence, social media usage patterns vary significantly in this age group. Moreover, minors were not included in the study, due to their contrasting interest in social media content. The aforementioned category of tech users accesses virtual channels to connect with friends and is inclined to spend the majority of their time online on TikTok, according to a 2022 Pew Research Center analysis [5].

**Figure 1:**
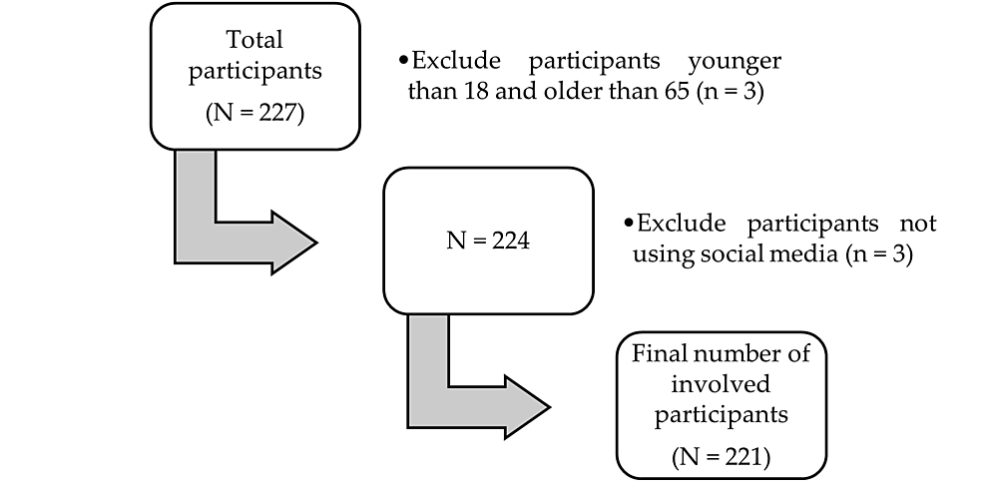
Flowchart of data selection

Facebook and Instagram platforms were chosen for the present study, because of the high number of Romanian users registered in early 2022. As per the statistics, among the Romanian population totaling 19.08 million inhabitants, 9.90 million people used Facebook, while 5.40 million Instagram users were identified [6]. Therefore, out of 227 people who completed the questionnaire in the two-month period of survey availability, 221 met the aforementioned criteria (Figure 1).

The survey was created and hosted by SurveyPlanet (Marina Del Rey, California, United States) and it included 20 questions, divided into four categories: (1) patients’ general characteristics, (2) social media usage types and opinions on its modifying role concerning melanoma prevention attitudes, (3) personal applicability of the correct sun protection behaviors and (4) data on the personal and family history of melanoma and dysplastic nevi. The first question category was designed in order to establish the demographic and epidemiological features of the participants and to facilitate the selection process. The second part of the quiz focused on the way each individual chooses to use social media channels (regarding preferences for specific platforms and interest in medical content); extracting this information was considered relevant due to its possible correlation with the data comprised in the third section of the questionnaire. The fourth category of questions was introduced with the aim of identifying whether the personal risk factor constellation or the family history of dysplastic nevi or melanoma, which theoretically expose these certain individuals to specialty medical examinations and counseling, have the ability to increase the understanding of sun protection importance, hence refining the personal good dermatological practices.

In the questionnaire, 14 questions were single-answer multiple choice questions, and three of these approached demographic characteristics, in order to gain better insight into the participants and their social backgrounds. Furthermore, two multiple-answer multiple-choice questions were included in the survey and were used to investigate the most common sun protection behaviors and the most frequent dermatological advice that people applied after seeing it online. These predetermined options were chosen for their appropriateness, as they contribute to a user-friendly survey-taking experience. The standardized phrasing of the response options facilitates the systematic collection of homogeneous data. Moreover, this approach ensures consistency in responses and simplifies the quantitative analysis of the gathered information. Four-rating scale questions were integrated, with the aim of obtaining evidence regarding the extent to which people engage in certain photoprotective behaviors. Participants were provided with context for the accuracy of their answers; i.e., explanations of the values included in the scale ranging from 1 to 10 (where 1 is not at all applicable and 10 is highly applicable) were provided. The respondents also benefitted from a visual representation of the scale, thus rendering the participation more interactive and increasing the chances of precise response submission.

The participation was voluntary, the criterion was supported by the presence of the Start button at the beginning of the e-survey, and the extraction strategy followed the principles of chain sampling, in order to gain various models from the general population. Each respondent self-reported information about their sun protection behavior.

The confidentiality of the participants was respected through the anonymous nature of the questionnaire and the type of question phrasing that did not permit direct or indirect traceability back to a certain individual. The e-questionnaire respected the utmost national and international norms, in compliance with the Declaration of Helsinki (2000). Moreover, the study was approved, before disseminating the questionnaires, by the Ethics Committee of “Ovidius” University of Constanta, Romania.

The information provided by the respondents was processed using Microsoft Excel, 2018, (Microsoft Corporation, Redmond, Washington, United States), especially with the help of formulas specific for “average,” “standard deviation,” “covariance,” and “correlation coefficient.”

## Results

The final database comprised 221 individuals with the sociodemographic characteristics depicted in Table 1. Most of the respondents were aged between 26 and 35 (47.1%, n=104), predominantly females (77.82%, n=172), with a female:male ratio of 3.5:1. The majority of respondents (71.94%, n=159) lived in urban areas with >200000 inhabitants, as opposed to 3.61% (n=8) that resided in rural regions. From the targeted social media users, 44.79% (n=99) declared that they actively followed medical educational content through virtual channels, while 42.53% (n=94) stated that they only watched it when it was suggested or sent to them by someone else. On the other hand, 12.66% refused to search for medical advice and good dermatological practice online, because they either believed it was irrelevant (3.16%, n=7) or had other more reliable sources (9.5%, n=21).

**Table 1:** Socio-demographic characteristics of the studied population

Socio-demographic parameter	Percentual value
Gender	
Male	22.18%
Female	77.82%
Age	
18-25	12.22%
26-35	47.06%
36-45	19.00%
46-55	16.74%
56-65	4.98%
Area of residence	
Urban > 200000 inhabitants	71.95%
Urban 50000-200000 inhabitants	14.48%
Urban < 50000 inhabitants	9.95%
Rural	3.62%

Firstly, participants were provided with common sun-protective habits and asked to select whatever applied to their daily routine. Most of them (80.99%, n=179) declared that they used SPF (sun protection factor) products, 54.29% (n=120) wore sunglasses specially equipped with UV (ultraviolet) filters, and 53.84% (n=119) avoided sun exposure between 10 AM and 4 PM. Concerning the use of clothing, 109 participants (49.32%) acknowledged that they wore head accessories such as wide-brimmed hats, caps, or sun umbrellas, while 30.31% (n=67) admitted to covering their skin with white, long-sleeved clothes. Only a minority of respondents (7.69%, n=17) confessed to not using any sun protective measures, and 3.61% (n=8) endorsed the adoption of UV-blocking clothes.

**Figure 2:**
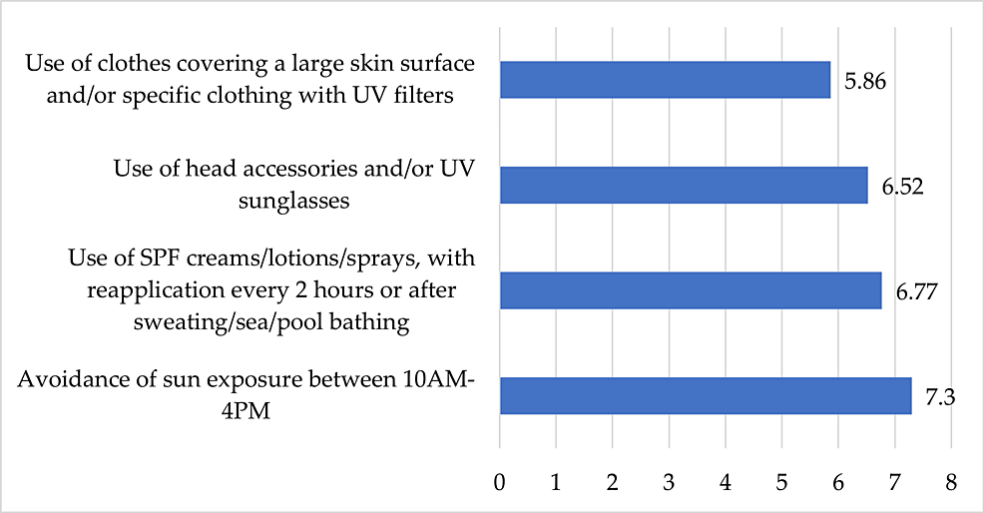
Sun-avoidance behaviors and average applicability value measured on a scale from 1 (not at all applicable) to 10 (highly applicable)

Consequently, respondents were requested to rate, on a scale from 1 (not at all applicable) to 10 (highly applicable), the extent to which a certain sun protective measure applied to their regime (Figure 2). Therefore, the mean value assigned to the use of SPF creams/lotions/sprays, with reapplication every two hours or after sweating/sea/pool bathing, was 6.77, and the use of clothes covering a large skin surface and/or specific clothing with UV filters was valued at 5.86. Moreover, the use of head accessories and/or UV sunglasses scored roughly 6.52. Avoidance of sun exposure between 10 AM and 4 PM reached the highest average value of 7.3.

**Figure 3:**
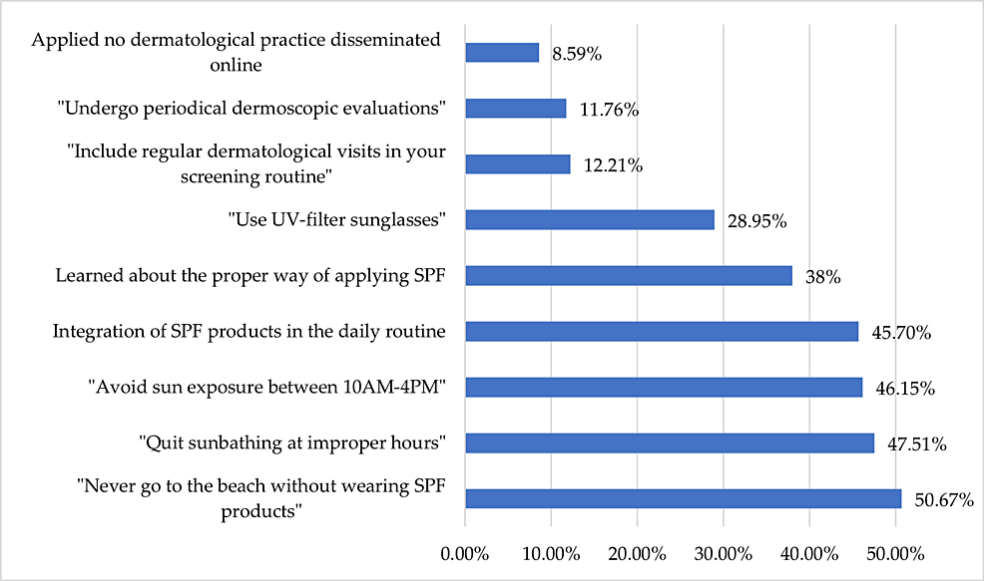
Most frequent social media-transmitted dermatological advice incorporated into the routine of the general population (n=221)

When asked about which dermatological advice transmitted on social media was incorporated into their routine, participants were provided with multiple options that defined the most frequent suggestions made by dermatologists in the digital environment, accounting for different degrees of responsibility and personal skin health commitment (Figure 3). In total, 112 individuals (50.67%) stated that they never went to the beach without wearing SPF products, 102 (46.15%) quit sunbathing at improper hours, and 101 (45.7%) started avoiding sun exposure between 10 AM and 4 PM. A total of 47.51% (n=105) integrated the use of SPF products in their daily routine, while 38% (n=84) learned how to properly apply SPF, and 64 participants (28.95%) declared that they started using UV-filter sunglasses. Contrastively, 27 persons (12.21%) reported that they did not appropriate any dermatological practice disseminated online. One respondent stated they installed mobile applications that display the UV index.

As far as specific dermatological check-ups are concerned, 11.76% (n=26) of participants included regular dermatological visits in their screening routine, while 8.59% of them (n=19) started undergoing periodical dermoscopic evaluations as a result of social media independent or national campaigns.

A total of 17.2% (n=38) admitted to having used tanning beds in the past, 81% (n=179) had never been exposed to artificial tanning devices, while four participants (1.8%) were using tanning devices at the time of form completion, with a bimonthly frequency.

A question regarding the significance of the word melanoma was included in the e-questionnaire, in order to quantify the degree of dermatological education; therefore, the majority of respondents (85.52%, n=189) chose the meaning of skin cancer subtype as the correct answer, 9.95% of them (n=22) declared they did not know its significance, while 4.52% (n=10) selected the option “dermatological contagious disease” as the right response. 

**Figure 4:**
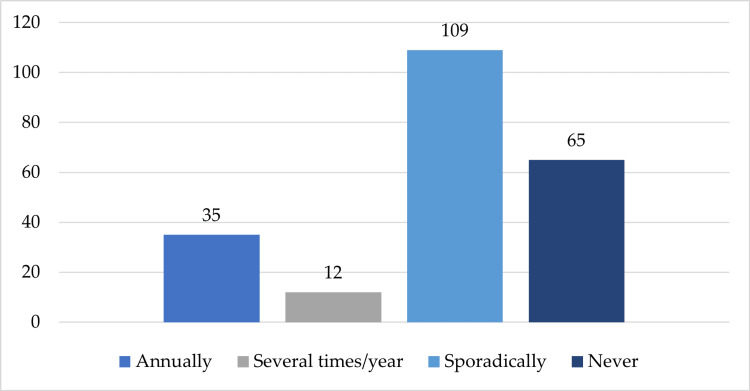
Frequency of dermatological check-ups in the investigated population (n=221)

In the third section, dedicated to the personal applicability of skin cancer screening approach, 109 interviewees (49.32%) had made dermatological appointments sporadically (once every several years), 65 of them (29.41%) had not undergone a specific regular check-up, while just 47 participants (21.26%) stated that they had visited a dermatologist annually or several times per year, according to their cutaneous characteristics and findings (Figure 4).

**Figure 5:**
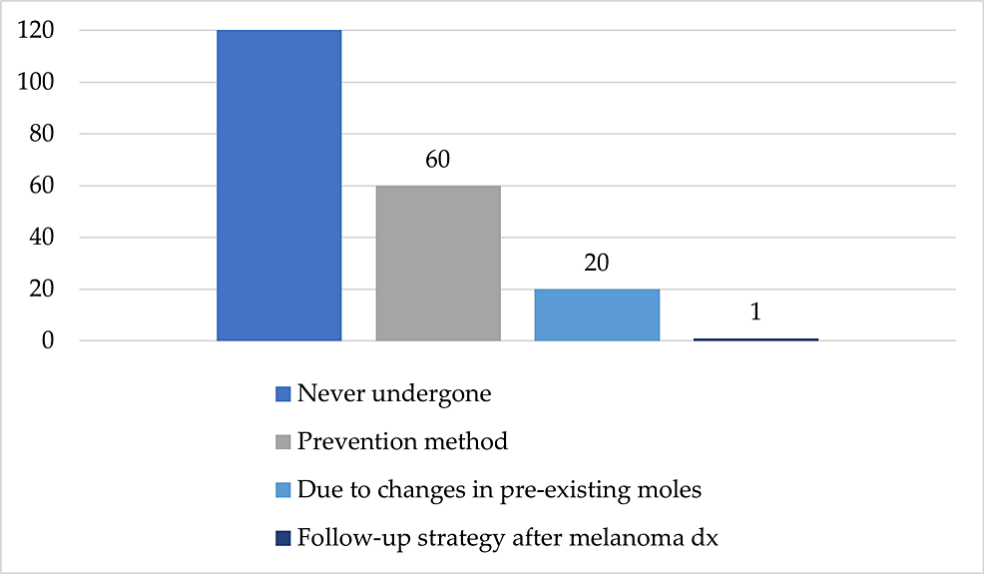
Frequency of dermoscopic evaluations in the investigated population (n=221)

When asked about their patient experience with dermoscopic evaluations, the vast majority (63.34%, n=140) disclosed they had never received a dermoscopic evaluation of their skin lesions; on the contrary, 27.14% (n=60) undertook it as a prevention method and 9.04% (n=20) requested it due to recent changes in the appearance of a pre-existing mole (Figure 5). 

Subsequently, regarding the personal and family history of dysplastic nevi, 2.26% (n=5) of the participants had been diagnosed with dysplastic nevi, while 1.35% of them (n=3) had first-degree relatives with the afore-described diagnosis.

Concerning the melanoma history, only three respondents originated in families with a member diagnosed with this disease, while one of them had a personal history of melanoma.

Eventually, the estimated survey completion length was seven minutes.

## Discussion

This is the first cross-sectional study performed among Romanian users of social media platforms that targeted the exploration of Internet-based feasibility of skin cancer awareness campaigns and dissemination of information regarding the screening of cutaneous oncological entities. In order to thoroughly assess the role of virtual channels in the propagation of the aforementioned facts, we chose Instagram and Facebook as the main publication sites for the link guiding the interested population to the e-questionnaire. During its 60-day period of activity, the survey was mostly accessed through Instagram, proving this platform’s leading part in the digital industry and its cost-effective, quasi bias-free potential of becoming an instrument of scientific data and advice reporting and propagation. As shown by Kim et al., followers of Instagram accounts run by dermatologists are mostly general population (66%) and medical students (10%), thus fortunately shifting the paradigm towards the propagation of exact and accurate healthcare knowledge to interested users [7].

Moreover, compared to the available information concerning selection bias encountered in typical randomised control trials (RCTs), this mean of questionnaire dissemination proved coherent in involving certain populational categories that are usually omitted, such as women [8]. The short amount of time needed to obtain 221 valid answers additionally pleads for the use of virtual channels to the detriment of conventional data collection forms or paper medical records [9].

The limitations of this study that need to be addressed are the collection of self-reported data that may be susceptible to hypothetical and recall bias, as well as the chain sampling method that may have implied selection bias. Supplementarily, elderly people or those originating from unfavorable environments, who do not possess technical skills and/or are not granted access to the Internet may be severely impacted by a prospective migration of health-related information exclusively in the digital area. 

In this analysis, the hypothesis according to which the higher applicability of dermatological recommendations disseminated on social media was corroborated with specific epidemiological traits of the participants was tested. In order to achieve this, the covariance and correlation coefficient were calculated. A positive covariance was interpreted as the expression of the identical direction that two variables follow, whereas negative covariance results were associated with divergent relationships between two concepts. Furthermore, a correlation coefficient of 1 was considered to denote a perfect positive relationship between any two variables, a coefficient above +0.75 or under -0.75 revealed a strong correlation, while negative values infirmed the suppositions.

Firstly, the association between the younger age (under 35) and more prominent adoption of good dermatological practices was confirmed, due to the positive values of the covariance determination (0.02-0.05) but could not be considered significant according to the correlation coefficients that varied between 0.09 and 0.23, which highlighted a weak correlation.

Secondly, the link between the urban place of residence, taking into consideration the more facile access to technology, and the higher acquisition of good dermatological practice was negligible, as shown by performing the covariance analysis (with results ranging from 0.005 to 0.008) corroborated with the correlation coefficients (0.05-0.08).

It appears that this particular type of e-survey does not only provide statistical data on specific dermato-oncological screening behaviors but can also act as an awareness-raising tool in regard to the proper sun protective approach and suitable dermatological referral frequency, especially when it encompasses science-based knowledge and is compiled by HCPs.

Moreover, as described by Attai et al., online platforms also represent a territory where patients who have already been diagnosed with a malignant condition may seek support, share their experiences, thoughts, and anxieties, and address their disease-related questions to fellow oncological patients [10]. Therefore, the importance of these virtual channels is not limited to the correspondence between the general population and HCPs, either specialists running personal accounts or doctors leading institutional platforms, but is augmented by the opportunity of acquiring psychological support from people who have been confronted with relatable medical experiences.

By corroborating the statistics from the United States that place melanoma as the third most prevalent malignancy among people aged 15-39 and the fact that more than 90% of people corresponding to this age bracket spend over nine hours daily online, it becomes clear that the main targeted audience should be composed of adolescents and young adults [11,12]. The present study is consistent with the data found in the literature because most of the e-survey respondents were between 26-35 years old. However, the results concerning the association between a higher adoption of sun-protective behaviors communicated via social media and a greater amount of time spent online daily (using the young age and urban place of residence as proxies) highlighted the fact that these concepts are weakly interconnected. Therefore, additional studies investigating the characteristics of the target audience with a higher addressability of dermatological advice are suggested. The criteria that can be explored in the future as influential to the design of skin cancer awareness campaigns are socioeconomic status, educational degrees, marital status, the potential impact of parenthood, and the professions of the participants. 

Furthermore, the epidemiological information extracted from the questionnaire, depicting the prevalence of female responders, may constitute an important parameter for the design of skin cancer awareness campaigns. Female users tend to respond better to visual representations, while men do not perceive online content creators as trustworthy as women do in the absence of scientific proof [5,13].

Since social media usage styles are constantly changing and due to the incessant migration of users between applications according to emerging trends, dermatologists should take into consideration the appropriate online landscape for the unfolding of awareness strategies. For example, Facebook and Twitter lost their leading place among teen users after the emergence of Instagram and TikTok [11]. Therefore, skin cancer prevention information may be utterly distributed and assimilated by both sexes if the campaigns used visual elements displaying the effects of unprotected sun exposure (such as images generated through UV light cameras that assess the degree of melanin accumulation on the most prominent facial areas or clinical pictures of melanoma lesions), in order to target the female population, in conjunction with science-based explanations dedicated to the male audience. Moreover, skin cancer awareness initiatives should deliver brief and condensed video content, taking into consideration the perpetual shortening of social media users’ attention span, which decreased from 12 to eight seconds per piece of content, compared to 20 years ago [14]. These concise posts may be accompanied by links directing the reader toward more detailed articles or by captions exhaustively approaching the particular matter. 

The value of the online environment as a melanoma prevention tool should, however, be restricted to primary prevention and the dissemination of important basic sun-avoidance behaviors because it cannot substitute the personalized recommendations given by dermatologists in the examination room. Dermatological consultations provided via social media should not be endorsed, either, due to the low probability of clinical accuracy delivered by non-professional pictures of the cutaneous organ, the incomplete information regarding patients’ skin particularities and comorbidities, and the lack of dermoscopic assessment.

## Conclusions

This study provides insights into the dermatological behaviors and education of individuals engaging with social media medical content. The results suggest that constant users of social media harbor a receptive attitude toward dermatological advice provided on specific platforms, hence creating an ideal environment for further dissemination and organization of online skin cancer awareness campaigns. However, taking into account the low proportion of people who currently schedule regular dermatological visits and dermoscopic examinations, it is clear that more educational programs on this matter should be implemented, in order to catch the attention of the general population. Additional cross-sectional population-based studies are required to detect the appropriate techniques and means of propagating the correct melanoma and non-melanoma skin cancer (NMSC) prevention measures for targeting large cohorts and establishing early diagnosis, thus reducing the morbidity and mortality rates associated with these oncological entities.
